# Efficacy and safety of drug-coated balloon in the treatment of acute myocardial infarction: a meta-analysis of randomized controlled trials

**DOI:** 10.1038/s41598-022-10124-z

**Published:** 2022-04-21

**Authors:** Qiu-Yi Li, Mei-Ying Chang, Xin-Yi Wang, An-Lu Wang, Qi-Yu Liu, Tong Wang, Hao Xu, Ke-Ji Chen

**Affiliations:** 1 National Clinical Research Center for Chinese Medicine Cardiology, Xiyuan Hospital, China Academy of Chinese Medical Sciences, Beijing, 100091 China; 2 Beijing University of Chinese Medicine, Beijing, 100029 China

**Keywords:** Cardiology, Interventional cardiology

## Abstract

Acute myocardial infarction (AMI) is one of the main causes of death in the world, and the incidence of AMI is increasing in the young population. Drug-coated balloon (DCB) has become an effective concept for the treatment of in-stent restenosis, small vessel disease, bifurcation lesions, high blood risk conditions, and even de novo large vessel disease. To ensure whether DCB can play an alternative role in AMI, we conducted a comprehensive meta‐analysis of randomized controlled trials (RCTs) to evaluate the efficacy and safety of DCB in the treatment of AMI. Electronic databases were searched for RCTs that compared DCB with stent for AMI. The primary outcome was major adverse cardiac events (MACEs), the secondary outcome was late lumen loss (LLL). RevMan 5.3 software and RStudio software were used for data analysis. Five RCTs involving 528 patients with 6–12 months of follow-up were included. There was no significant difference in the incidence of MACEs between DCB group and stent group (RR, 0.85; 95% CI 0.42 to 1.74; *P* = 0.66). Lower LLL was shown in DCB group (WMD, − 0.29; 95% CI − 0.46 to − 0.12; *P* < 0.001). This meta-analysis of RCT showed that DCB might provide a promising way on AMI compared with stents. Rigorous patients’ selection and adequate predilation of culprit lesions are necessary to optimize results and prevent bailout stent implantation.

*PROSPERO registration number*: CRD42020214333.

## Introduction

Acute myocardial infarction (AMI) is one of the main causes of death in the world, and the incidence of AMI is increasing in the young population^1^. Early myocardial reperfusion through medication, surgery or intervention is the main treatment for AMI^2^. Compared with bare-metal stent (BMS), new-generation drug-eluting stent (DES) reduces the incidence of target vessel revascularization and stent thrombosis, and is therefore recommended for the treatment of patients with AMI in 2021 ACC/AHA/SCAI Guideline for Coronary Artery Revascularization^3^. However, stent-related complications, such as recurrent myocardial infarction (MI) and in-stent restenosis, may recur several years after stenting, and bleeding complications from dual antiplatelet therapy (DAPT) after stenting should not be ignored, and stenting may not reduce the mortality or recurrence rate of MI compared with balloon angioplasty alone^4^. After more than a decade of research, drug-coated balloon (DCB) has become a new concept for the treatment of coronary heart disease (CHD), and is increasingly used especially because it can play a unique role in the treatment of in-stent restenosis (ISR), avoiding the overlap of multiple layers of stents. Many clinical trials have also demonstrated its value in small vessel disease, bifurcation lesions, high blood risk conditions, and even in de novo large vessel disease^5^. DCB can rapidly and uniformly transfer the anti-proliferative drugs attached to its surface to the vessel wall of the lesion site by balloon dilation, thus alleviating or relieving the stenosis without the use of permanent implants and inhibiting the proliferation of endothelial cells^6^. Although a number of recent clinical trials have evaluated the feasibility of DCB for the treatment of AMI patients, these individual studies do not provide very strong evidence of the exact efficacy of DCB for AMI^7–9^. To ensure whether DCB can play an alternative role in AMI, we conducted a comprehensive meta‐analysis of randomized controlled trials (RCTs) to evaluate the efficacy and safety of DCB in the treatment of AMI.

## Materials and methods

### Systematic search and study selection

This study was performed according to the Preferred Reporting Items for Systematic Reviews and Meta‐Analysis (PRISMA) guidelines (Supplemental Table 1. PRISMA 2020 checklist)^10^, and was prospectively registered with the PROSPERO registry (CRD42020214333). No additional ethical clearance is required since this study is based on a secondary literature analysis of published RCTs. A systematic search was conducted in PubMed, Embase, Cochrane Library, Web of Science, Chinese National Knowledge Infrastructure (CNKI), Wanfang Database and Weipu Database without any language restrictions from their inception to November 2020. The major search terms were as follows: drug-coated balloon, myocardial infarction. We also conducted a manual search to confirm the relevant references in the selected articles. The search strategy used for PubMed was presented in Supplemental Table 2 and modified to suit other databases.

Clinical trials that met the following criteria would be included in this study: Participants were AMI patients aged ≥ 18 years old; Interventions for culprit vessels were DCB-only procedures or stenting (either BMS or DES); Participants in each study were followed for at least six months; RCTs. Diagnosed with ISR would be excluded from this study.

The primary endpoint was major adverse cardiac events (MACEs), defined as the composite of cardiac death, MI, and target lesion revascularization (TLR). The secondary endpoint was late lumen loss (LLL), obtained by calculating the difference between the minimum lumen diameter between follow-up and post-procedure.

### Data extraction and quality assessment

Two reviewers (AW and XW) independently extracted the data from the included studies, using a predetermined collection form that includes: demographic and lesion characteristics of the population of interest, selection criteria, interventions, study design, duration of follow-up, and clinical outcome data of interest. Clinical data would be extracted over the maximum available follow-up period. Disagreements, if any, would be resolved through discussion with the third author (HX). The risk of bias of eligible studies would be assessed by the Cochrane Collaboration's Risk of Bias tool, which consists of following 7 points: generation of the random allocation sequence, concealment of the allocation sequence, blinding of participants and physicians, blinding of outcome assessment, incomplete data, selection of reporting and other sources of bias^11^. Studies will be classified as low, high, or unclear risk.

### Data synthesis and statistical analysis

RevMan 5.3 software and RStudio software were used for data analyses. We calculated the weighted mean difference (WMD) with the corresponding 95% confidence interval (CI) for continuous outcomes and the risk ratio (RR) with its 95% CI for dichotomous data. The I^2^ statistic and Cochran’s Q test were used to test statistical heterogeneity. Cochran’s Q test *P* < 0.05 and I^2^ > 50% indicated the presence of heterogeneity in the relevant statistics. Results for which heterogeneity existed were analyzed using a random effects model, otherwise a fixed effects model was used to pool effect sizes.

If necessary, analysis of results for which heterogeneity exists will be performed using sensitivity analysis by examining the effect of excluding each study separately. If differences in outcomes are produced, factors that may contribute to heterogeneity will be analyzed, including but not limited to overall coronary artery disease status, target vessel caliber, treatment method, and type of stent in the stent group. P values less than 0.05 are considered statistically significant differences.

## Results

### Description of included studies

**Figure 1:**
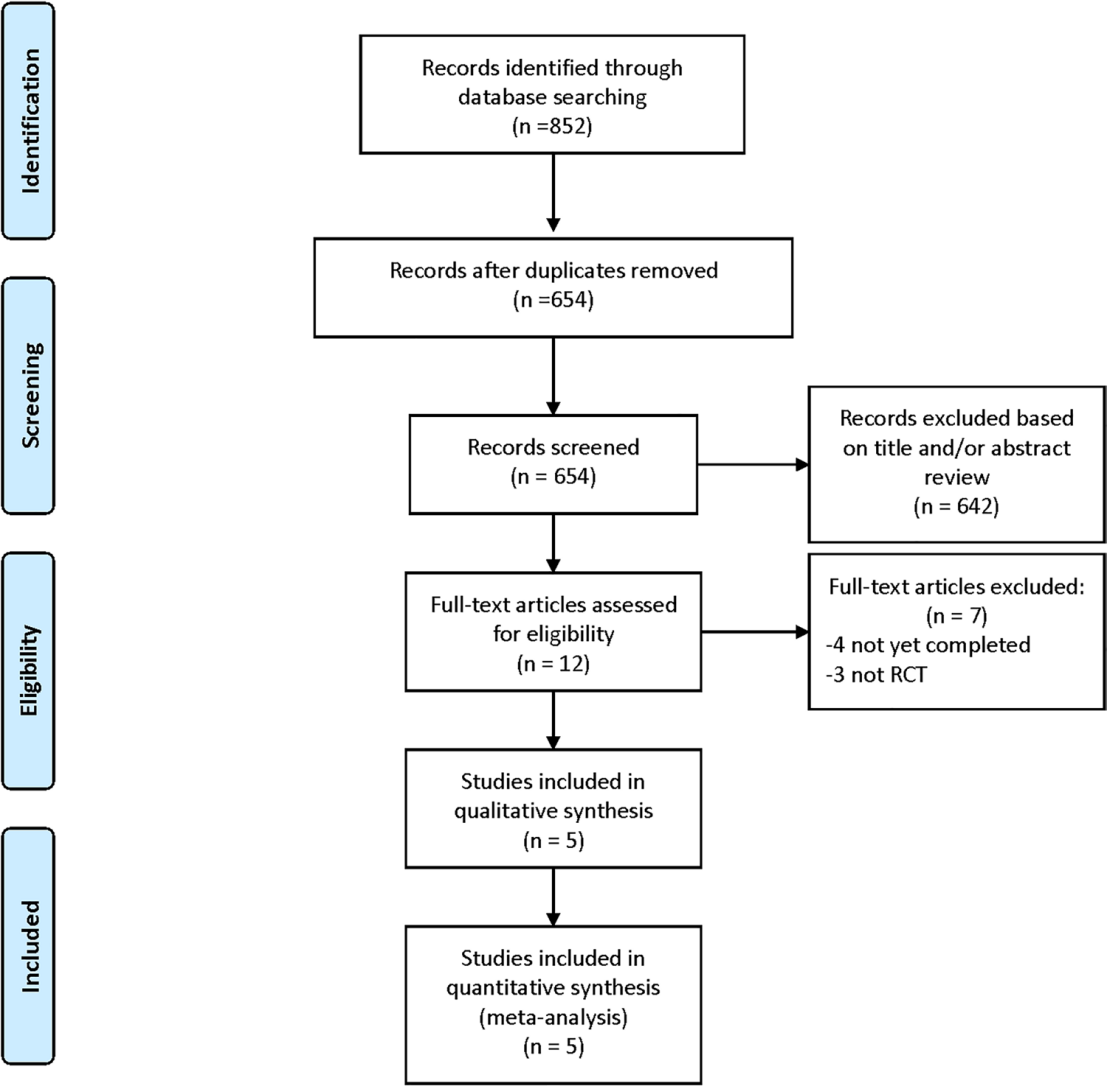
Study search diagram.

A total of 852 studies were identified through the electronic database search. Among them, 198 records were removed on account of duplicates, 642 records were excluded after screening of titles and abstracts. We filtered 12 articles requiring full-text screening, of which 5 RCTs fulfilled the eligibility criteria and were finally selected for the meta-analysis^8,9,12–14^. Details of study selection process were generated according to the PRISMA requirements (Fig. 1).

The characteristics of included trials are summarized in Table 1. The present analysis comprises 528 patients (DCB n = 252; stent n = 276). Baseline data for both groups of patients in each study were comparable. Procedural characteristics are presented in Table 2. Bailout stenting was advised only in case of residual stenosis or occurrence of clinically significant dissection. The bailout stenting rate of each study is from 5.7% to 18%. All patients received 1 year of DAPT, except one study in which patients in the DCB group received 6 months of DAPT after intervention (Table 2).

**Table 1:** Characteristics of the included studies.

Author, year	Country	Number of patients (DCB/stent)	Age(years) (DCB/stent)	Male (%) (DCB/stent)	BMI (kg/m^2^) (DCB/stent)	Presentation	Outcomes	Follow-up (months)
Gobić, 2017^12^	Croatia	38/37	56.6 ± 13.2 54.3 ± 10.6	71.1 73.0	29.4 ± 4.1 28.2 ± 3.7	STEMI	CD, MI, TLR, ST, LLL	6
Liu, 2020^14^	China	33/32	NA	NA	NA	STEMI	CD, MI, TLR, ST, LLL	12
Scheller, 2020^9^	Germany	104/106	66.0 ± 11.4 67.0 ± 13.1	66.3 67.9	28.7 ± 5.2 28.4 ± 4.9	NSTEMI	CD, MI, TLR, ACM, stroke, PCI in other vessels	9
VOS, 2019^8^	Netherlands	60/60	57.4 ± 9.2 57.3 ± 8.3	87 87	26.7 ± 3.5 27.4 ± 4.4	STEMI	CD, MI, TLR, FFR	9
Wang, 2020^13^	China	38/42	59 ± 13 56 ± 14	79 83	26 ± 7 25 ± 10	STEMI	CD, MI, TLR, LLL	12

*DCB* drug-coated balloon, *BMI* body mass index, *STEMI* ST-segment elevation myocardial infarction, *NSTEMI* non-ST-segment elevation myocardial infarction, *CD* cardiac death, *MI* myocardial infarction, *TLR* target lesion revascularization, *ST* stent thrombosis, *LLL* late lumen loss, *ACM* all-cause mortality, *PCI* percutaneous coronary intervention, *FFR* fractional flow reserve.

**Table 2:** Procedural Characteristics.

Author, year	Premedication	Lesion preparation	DCB type	Stent type	Bailout stenting rate (%)	DAPT (months) (DCB/stent)
Gobić, 2017^12^	aspirin plus clopidogrel	Thrombus aspiration and/or balloon dilation	Sequent Please	Cobalt-chromium sirolimus eluting stents	7.3	12/12
Liu, 2020^14^	aspirin plus ticagrelor	Balloon dilation	Sequent Please	Xience V DES	5.7	12/12
Scheller, 2020^9^	aspirin plus clopidogrel, ticagrelor or prasugrel	Balloon dilation	Sequent Please	56%BMS and 44%DES	15	12/12
VOS, 2019^8^	aspirin plus ticagrelor or prasugrel	Thrombus aspiration and/or balloon dilation	Pantera Lux	Sirolimus or Everolimus (Orsiro, Biotronik; or Xience, Abbott, Abbott Park, Illinois)	18	12/12
Wang, 2020^13^	aspirin plus clopidogrel	Thrombus aspiration and/or balloon dilation	Sequent Please	DES	9.5	6/12

*DCB* drug-coated balloon, *DES* drug-eluting stent, *BMS* bare-metal stent, *DAPT* dual antiplatelet therapy.

### Risk of bias assessment of included studies

**Figure 2:**
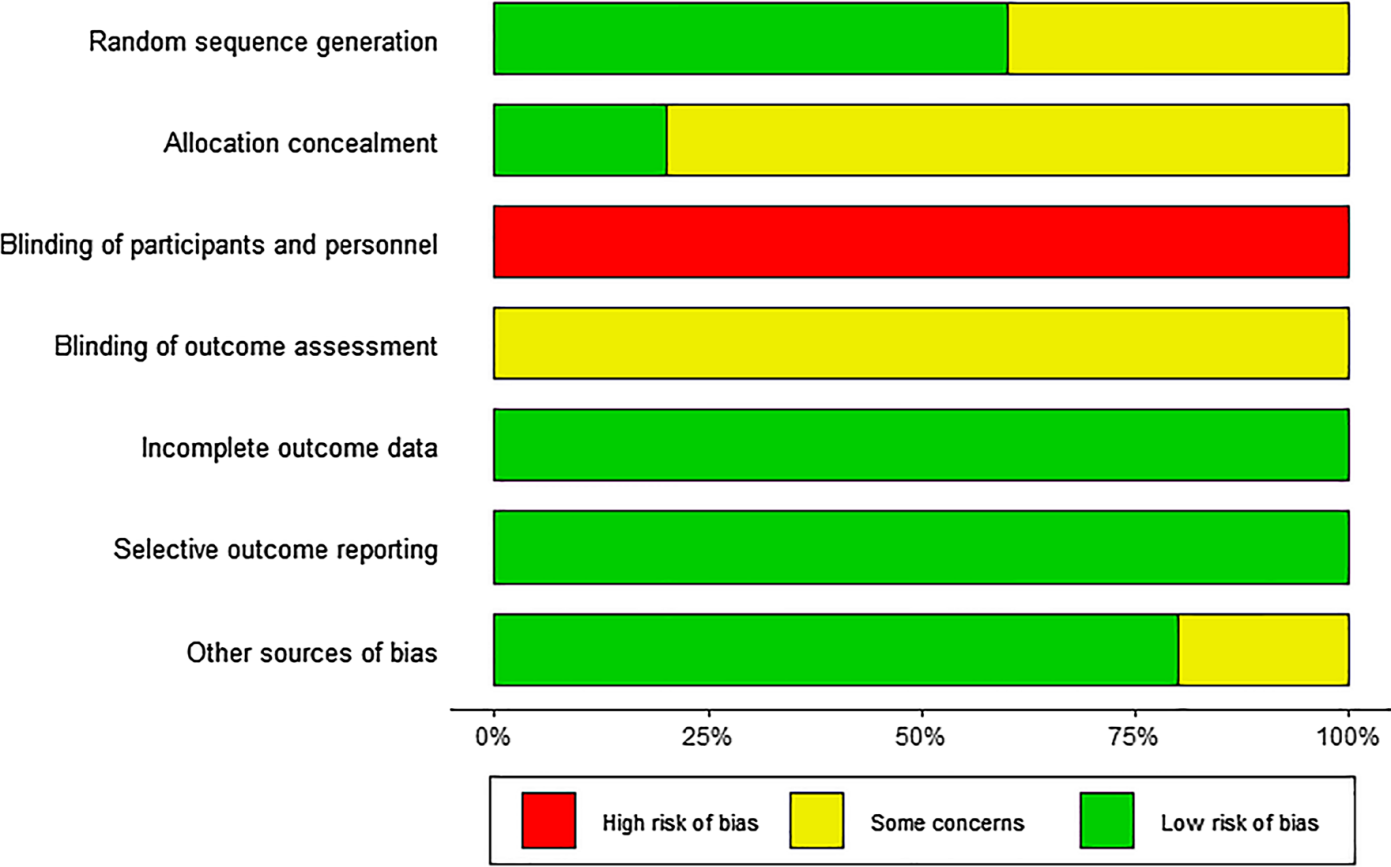
Risk of bias graph.

The results of the risk of bias assessment are shown in Fig. 2. The randomized assignment of participants was mentioned in all trials. Three of the trials illustrated methods of sequence generation, including computer-generated random numbers, random number table, and electronic randomization systems. One trial mentioned the adequate allocation concealment. None of the trials used blinding methods since it is difficult to achieve it in interventional operations (Supplemental Table 3).

### Primary outcome

**Figure 3:**
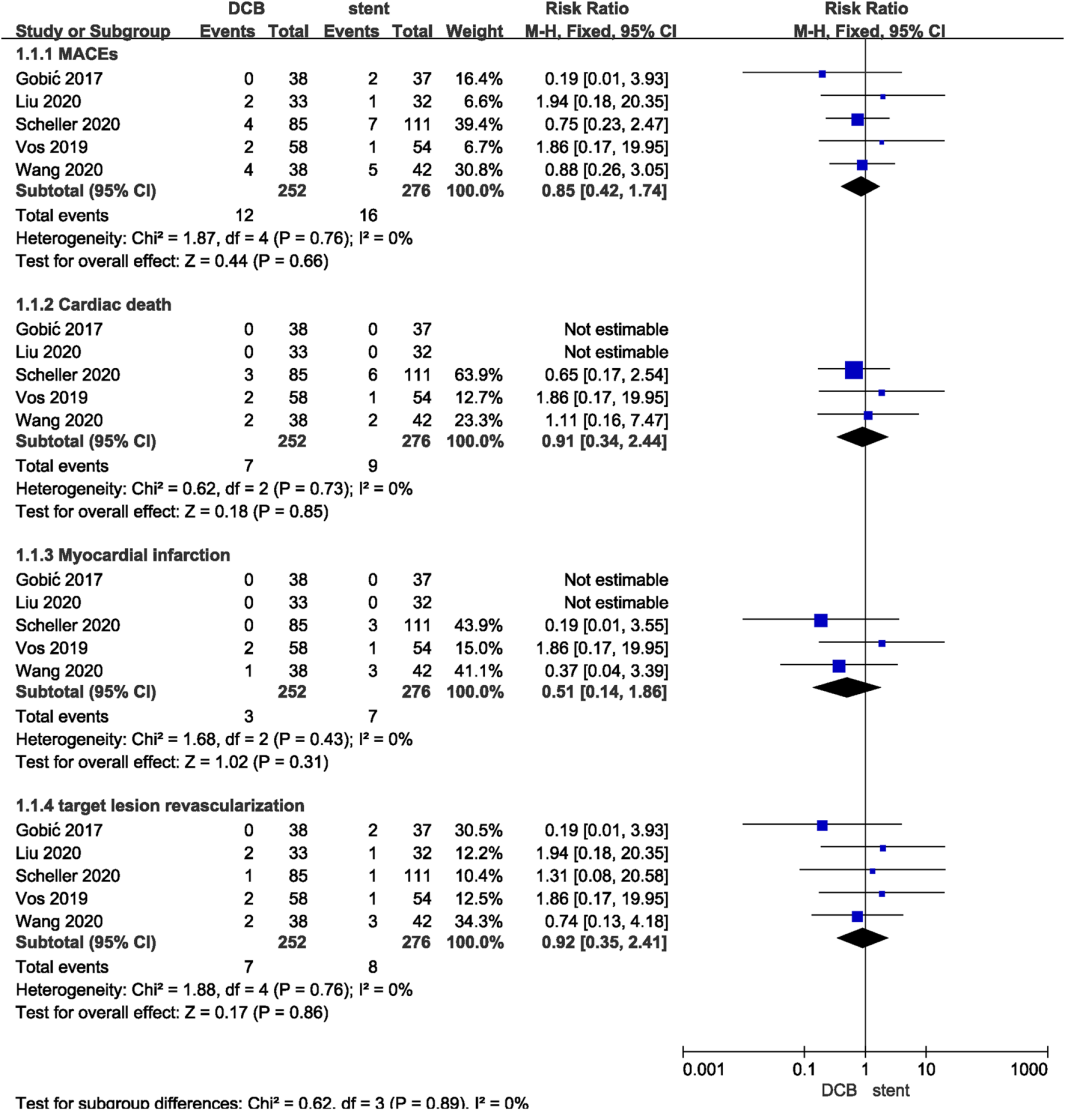
Risk Ratio (RR) of major adverse cardiac events (MACEs).

All five included studies reported the incidence of MACEs. Follow-up time of the studies ranged from 6 to 12 months. There was no significant difference in the incidence of MACEs between DCB group and stent group (RR, 0.85; 95% CI 0.42 to 1.74; *P* = 0.66). Individual analyses of each component of MACEs, including cardiac death, MI, and TLR, did not show significant differences (Fig. 3).

### Secondary outcomes

**Figure 4:**
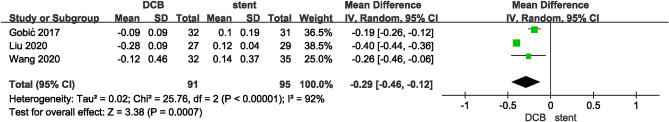
Risk Ratio (RR) of late lumen loss (LLL).

Three studies compared LLL between DCB group (n = 91) and stent group (n = 95). Compared with stent group, lower LLL was shown in DCB group (WMD, − 0.29; 95% CI − 0.46 to − 0.12; *P* < 0.001), suggesting that DCB may lead to positive coronary lumen remodeling. Sensitivity analysis was consistent with the primary analysis (Fig. 4).

### Subgroup analysis

**Figure 5:**
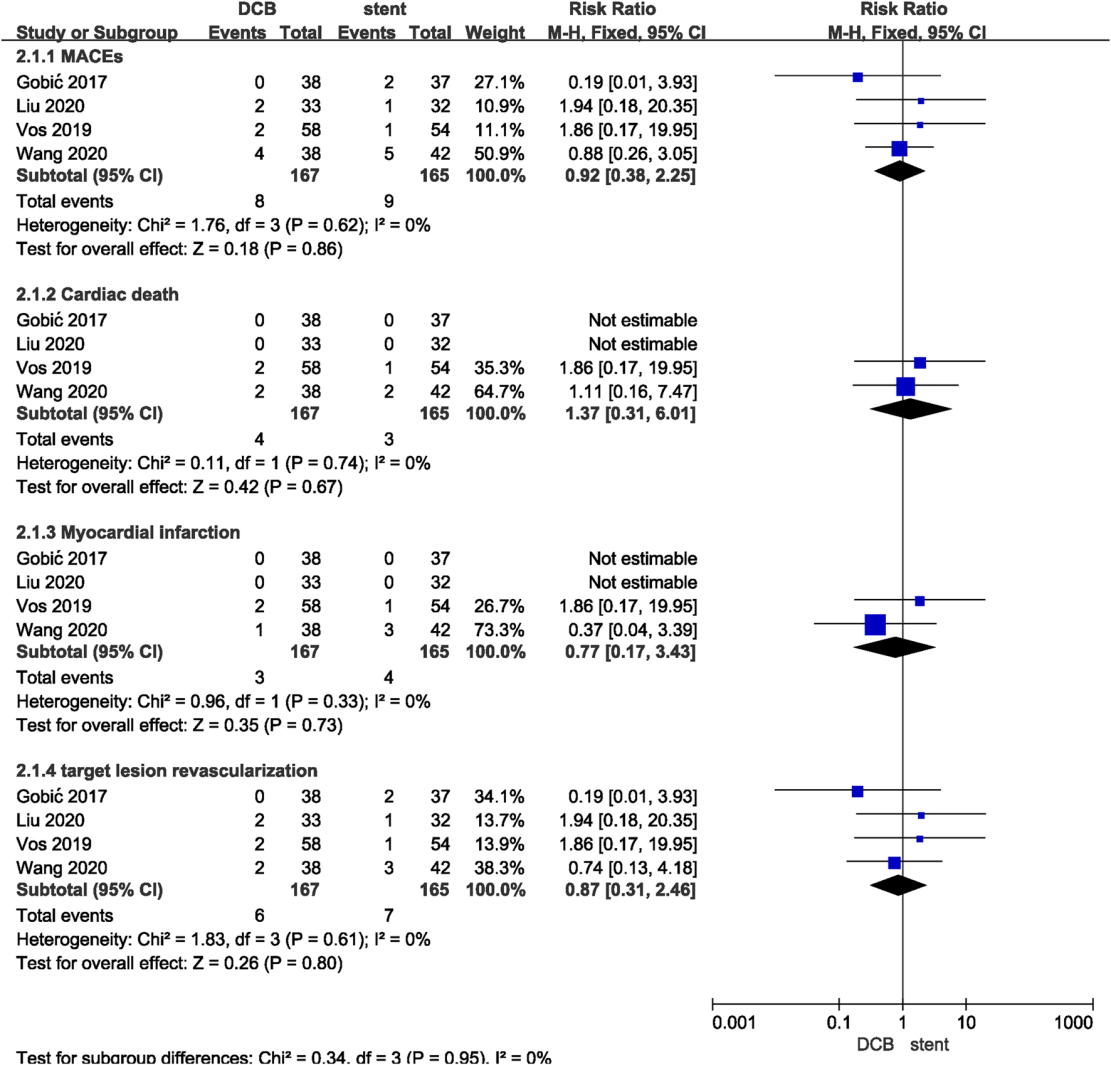
Subgroup analysis in STEMI.

Subgroup analyses were performed to evaluate the effect of DCB in patients with ST-segment elevation myocardial infarction (STEMI). There were no significant differences in MACEs, cardiogenic death, MI, or TLR between the two groups (*P* > 0.05) (Fig. 5).

## Discussion

This meta-analysis included 5 RCTs involving 528 patients with AMI undergoing DCB-only or stent implantation. The results indicated that DCB presented no significant difference in MACEs, cardiac death, MI and TLR for AMI compared with stents, whereas LLL was smaller in DCB group. We performed a subgroup analysis and could observe that among STEMI patients, the incidence of MACEs in the DCB group was similar to that in the stent group. Thus, these data indicated that DCB might provide a promising way for AMI.

In recent years, more and more studies have been conducted on DCB. In the treatment of ISR, meta-analysis of 10 RCTs from the DAEDALUS study showed that the composite incidence of all-cause death, MI, or target lesion thrombosis was similar for DCB treatment versus re-stenting, but DES repeat stenting was moderately more effective than DCB angioplasty in reducing the need for TLR at 3 years^15^. In a meta-analysis for the treatment of small vessel disease, the application of DCB was associated with comparable outcomes of MACEs when compared with DES^16^. Similarly, in patients with de novo coronary lesions, the use of DCB is associated with comparable clinical outcomes, such as TLR, compared with DES^17^. DCB has shown safety and efficacy in the treatment of various types of coronary lesions, and has prompted thinking about its use in AMI.

AMI is a common cardiac emergency that can lead to severe morbidity and mortality. The management of AMI has improved dramatically over the past three decades and is evolving^18^. Unlike the treatment of older AMI patients, the increased prevalence of AMI in younger people has forced us to pay attention to the long-term risks after stenting, such as lifelong medication, bleeding, etc^19^. DCB can delivers antiproliferative drugs locally without metal support, thereby directly inhibiting the process of endothelial proliferation and negative remodeling. The advantages of treatment with DCB dilation over DES implantation include a lower incidence of restenosis, shorter DAPT time to reduce the risk of bleeding, and the ability to promote further recovery of endothelial function without leaving any metallic material in the vessels^20^.

In this study, lower LLL and even extensive lumen enlargement were found in DCB group. It seems to indicate that DCB can result in positive coronary remodeling. Positive remodeling after DCB in de novo lesions was also reported by several studies^21–23^. The exact mechanism of late lumen enlargement is currently unknown and may be related to the long-term antiproliferative effects of drugs such as paclitaxel. Of course, the determination of lumen diameter in studies is mostly based on the results of coronary angiography, and more detailed and accurate assessment of lumen size and plaque regression by intra-luminal imaging such as intravascular ultrasound or optical coherence tomography is needed in the future^21^. To further confirm this conclusion, longer follow-up observations are needed.

DCB as an attractive “leave nothing behind” strategy may be safe and effective for the treatment of AMI. From another point of view, we should be cautious about the rate of bailout stenting ranging from 5.7% to 18%. This is often due to inadequate predilation of the lesion, resulting in elastic retraction or severe dissection of the vessel wall after DCB angioplasty, which necessitates the use of stents. It should be emphasized that use of DCB for AMI is based on the safe and effective predilation of culprit lesion. To derive maximum benefit from DCB, adequate predilation, especially in calcified lesions, is essential to maximize the contact area between the balloon and the vessel wall^24^.

Coronary calcification is an important factor affecting the prognosis of patients with CHD, and the occurrence of calcification is related to factors such as advanced age, chronic kidney disease, and diabetes^25,26^. Severe calcification leads to decreased stent expansion rate, more likely to trigger ISR and TLR, and is associated with MACEs^26–28^. Treatment strategies for calcified lesions require careful consideration. Data on the treatment of calcified lesions were lacking in the studies included in this meta-analysis. Devices such as cutting and scoring balloons, rotational atherectomy, laser coronary atherectomy are used for the treatment of severe calcified lesions, and study also showed that there was no significant difference in 1-year MACEs between DCB and DES after rotational atherectomy^29^. However, rotational atherectomy and laser coronary atherectomy may lead to increased operative time. It may be more reasonable to pretreat the lesion with a common balloon or a cutting and scoring balloon to achieve coronary reperfusion in a short period of time in MI patients^26^. The treatment strategy of MI complicated with calcified lesions needs further study.

In addition, the RCTs included in this study indicated that thrombus aspiration was performed on lesions with large thrombotic burden. Although routine thrombus aspiration did not affect mortality in trials^30^, researchers noted that optimizing the preparation of lesions was of great value in improving homogeneous delivery of antiproliferative drugs^8^.

In general, adequate preparation of lesions, including thrombus aspiration and adequate balloon dilation, is essential for DCB or stent therapy. For lesions with residual stenosis less than 30% or type A or B dissection, either DCB or DES can be used. DCB may be beneficial for younger patients with STEMI or those who are at increased risk of bleeding and in case of intolerance for DAPT. Bailout stenting was advised in case of residual stenosis of the treated lesion > 50% after dilatations with sufficiently large balloons, or coronary dissection greater than or equal to type C leading to vessel closure^8^.

Our meta-analysis has several limitations. First, the follow-up time for the five included studies was 6 to 12 months. The shorter follow-up time does not provide a good indication of the advantages of a DCB strategy that does not require long-term antiplatelet therapy, nor does it demonstrate its long-term safety. Although the results of the 5-year follow-up study demonstrated the safety of DCB for the treatment of de novo coronary artery disease, the long-term efficacy of DCB for AMI needs to be further investigated^31^.

Second, the sample size may be too small. However, we have conducted a very comprehensive literature search to include all articles that met the criteria. On the other hand, our study may also provide theoretical support for more investigators to conduct such studies in the future.

Third, we observed heterogeneity in the statistics of LLL. According to the study design, we adopted a random-effect model to estimate the effect rather than a fixed-effect model, because the former measures provided more conservative results^32^. The same result was obtained after sensitivity analysis.

Overall, this is the first meta-analysis of RCTs comparing DCB angioplasty with stenting for AMI, which can provide new ideas and thoughts for some interventional operators to treat AMI in the future, promote the development of interventional techniques, and improve the long-term prognosis of AMI patients. Further extended researches are supposed to support our findings.

## Conclusion

This meta-analysis of RCT showed that DCB might provide a promising way on AMI compared with stents. Rigorous patients’ selection and adequate predilation of culprit lesions are necessary to optimize results and prevent bailout stent implantation. More high-quality RCTs and longer follow-up are needed to confirm this conclusion.

## Supplementary Information

Supplementary Tables.

## Data Availability

All relevant data supporting the conclusions of this study are included in the article.
